# Tuina for chronic fatigue syndrome: study protocol for a randomized controlled trial integrating tryptophan metabolism, gut microbiota, and brain network alterations

**DOI:** 10.3389/fnins.2026.1859226

**Published:** 2026-08-12

**Authors:** Ming Jin, Sitong Fang, Shanda Xu, Ben Cao, Jinghu Li, Jun Ren, Shoujian Wang, Lingjun Kong, Qingguang Zhu, Min Fang

**Affiliations:** 1 Shuguang Hospital, Shanghai University of Traditional Chinese Medicine, Shanghai, China; 2 Institute of Tuina, Shanghai Institute of Traditional Chinese Medicine, Shanghai, China; 3 Yueyang Hospital of Integrated Traditional Chinese and Western Medicine, Shanghai University of Traditional Chinese Medicine, Shanghai, China

**Keywords:** chronic fatigue syndrome, gut microbiota, gut-brain axis, magnetic resonance spectroscopy, resting-state fMRI, tryptophan metabolism, Tuina

## Abstract

**Background:**

Chronic fatigue syndrome (CFS) is a disabling multisystem disorder characterized by persistent fatigue, post-exertional malaise, unrefreshing sleep, and neurocognitive and gastrointestinal symptoms. Effective non-pharmacological treatments remain limited. Increasing evidence suggests that CFS involves not only peripheral metabolic and gastrointestinal abnormalities but also central nervous system dysfunction. Altered tryptophan metabolism and gut-brain axis dysfunction may contribute to its pathophysiology. Tuina, a manual therapy used in traditional Chinese medicine, has been hypothesized to influence somatosensory, autonomic, stress-related, and gastrointestinal pathways. However, randomized controlled trials evaluating its clinical effects and exploratory gut-brain correlates in CFS remain scarce.

**Methods:**

This multicenter, parallel-group, randomized controlled trial will enroll 166 participants with CFS, who will be allocated in a 1:1 ratio to a Tuina group or a standardized usual care group. The Tuina group will receive treatment twice weekly for 8 weeks, whereas the control group will receive standardized usual care consisting of health education and symptom-oriented management. The primary objective is to evaluate the effectiveness of Tuina on fatigue severity, assessed by the change in Fatigue Scale-14 (FS-14) score from baseline to week 8. Secondary outcomes include quality of life, sleep, pain, anxiety, depression, gastrointestinal symptoms, bowel function, and handgrip strength. Exploratory mechanistic outcomes include plasma tryptophan-pathway metabolites, gut microbiota profiling, resting-state functional magnetic resonance imaging, and magnetic resonance spectroscopy. Assessments will be conducted at baseline, weeks 4 and 8, and at 12 and 24 weeks after completion of treatment.

**Discussion:**

This study is designed to evaluate the clinical effects of Tuina in CFS while exploring a biologically informed gut-brain framework involving tryptophan metabolism, gut microbiota, and brain network alterations. To our knowledge, few randomized trials in CFS have prospectively integrated clinical, metabolomic, microbiome, and multimodal neuroimaging measures within a single protocol. The findings may help clarify whether a non-pharmacological intervention can modulate interconnected peripheral and central pathways relevant to fatigue and related symptoms.

**Clinical trial registration:**

https://itmctr.ccebtcm.org.cn/mgt/project/view/1990257056896122880, Identifier ITMCTR2025002204.

## 1 Introduction

CFS is a complex multisystem disorder characterized by persistent disabling fatigue, post-exertional malaise, unrefreshing sleep, and a range of neurocognitive and gastrointestinal symptoms (National Institute for Health and Care Excellence (NICE), 2021). The global prevalence varies by case definition and study design, roughly 0.1–0.8% or up to around 1% (Vester et al., 2026). These manifestations substantially impair daily functioning and quality of life and impose a considerable social and economic burden. Current management is largely supportive, and some activity-based approaches may be poorly tolerated by patients who are prone to post-exertional symptom exacerbation. These considerations have renewed interest in low-burden, symptom-oriented interventions.

Although research on CFS has expanded in recent years, its pathophysiology remains insufficiently understood. Recent work increasingly supports a multisystem model of CFS involving peripheral metabolic, gastrointestinal, and central nervous system abnormalities. The 2021 NICE guideline no longer recommends graded exercise therapy based on fixed incremental increases in activity as a treatment for CFS, and it positions cognitive behavioral therapy as a supportive intervention to help patients manage symptoms rather than as a curative treatment (National Institute for Health and Care Excellence (NICE), 2021). In routine practice, symptomatic management is one of the main clinical approaches, yet many patients show little improvement in fatigue or post-exertional malaise even when care is consistent with current guidelines (Larun et al., 2024). In addition, activity-based interventions may be unsuitable for individuals who are prone to post-exertional symptom exacerbation. These limitations highlight the need to evaluate safe complementary and alternative therapies that may relieve symptoms and support physiological recovery in CFS. Patients commonly present with cognitive disturbance, pain sensitivity, autonomic dysregulation, and affective symptoms, and neuroimaging studies have reported altered functional connectivity across distributed large-scale brain networks in CFS (Inderyas et al., 2024). At the same time, growing evidence implicates gut microbial dysbiosis, altered microbial metabolites, and disturbed tryptophan-related pathways in symptom generation and persistence. These observations support the view that CFS involves abnormalities in distributed large-scale brain networks.

Non-pharmacological approaches are receiving increasing attention in CFS because some low-burden, symptom-oriented interventions may be more acceptable for patients with post-exertional malaise (PEM) (Wang et al., 2023; Li et al., 2024). Tuina is a structured manual therapy that delivers controlled tactile and pressure stimulation to defined body regions and acupoints (Ma et al., 2023). Preliminary studies suggest that Tuina may alleviate fatigue and improve gastrointestinal symptoms in patients with CFS (Li et al., 2017; Dai et al., 2023). More broadly, Tuina may exert multisystem effects through peripheral somatosensory stimulation, with potential downstream influences on autonomic regulation, stress responses, and gut-related function. Beyond its musculoskeletal effects, it may also enhance parasympathetic activity, modulate hypothalamic–pituitary–adrenal axis responses, and influence gastrointestinal motility (Geng et al., 2020; Packheiser et al., 2024).

At the same time, gastrointestinal symptoms are common in CFS, and growing evidence suggests that gut microbial composition, microbial metabolites, barrier-related processes, and immune signaling may be altered in this population (El-Sehrawy et al., 2025). In parallel, manual therapy applied to the abdomen and adjacent regions has been proposed to influence gastrointestinal motility and related visceral function, while other manual therapy studies suggest that such interventions may also modulate autonomic activity, including vagal responses (Goldenberg and Kalichman, 2024; Henley et al., 2008). Taken together, these observations raise the possibility that Tuina could affect gut-related physiological processes relevant to the gut-brain axis, including tryptophan metabolism, although these mechanisms remain to be clarified (Groven et al., 2021; König et al., 2022). However, rigorous randomized trials evaluating Tuina as an intervention for CFS remain scarce, and the mechanistic links among tryptophan metabolism, gut microbiota composition, and brain network function are still poorly defined. Importantly, these central abnormalities may not be independent of peripheral biological disturbances, particularly those involving immune signaling, gut microbial metabolism, and neuroactive metabolic pathways. In this context, tryptophan metabolism may provide a biologically plausible link between gut microbial alterations and central nervous system dysfunction, because tryptophan-derived metabolites can influence immune signaling, neuroactive pathways, and brain function. Therefore, an integrative design combining metabolomic, microbiome, and neuroimaging measures may help clarify whether peripheral and central abnormalities in CFS are mechanistically connected. Within this framework, tryptophan metabolism is considered a potential biological interface connecting gut microbiota-related alterations with central nervous system functional changes. Accordingly, microbiome and neuroimaging assessments are incorporated as complementary approaches to contextualize peripheral and central biological responses associated with clinical improvement following Tuina intervention.

In summary, Tuina may represent a low-risk and adaptable option for CFS, but its therapeutic effects and underlying mechanisms remain insufficiently characterized. This trial is designed to evaluate the efficacy of Tuina for reducing fatigue and improving related symptoms in patients with CFS. Gastrointestinal symptoms will be assessed as an important clinical and mechanistic domain rather than as an entry criterion. In addition, multimodal assessments, including functional MRI, magnetic resonance spectroscopy, metabolomic analysis, and metagenomic profiling, will be used to explore potential gut-brain mechanisms, with particular attention to associations among gut microbial features, tryptophan-related metabolites, and brain functional characteristics in CFS.

## 2 Objectives and hypotheses

### 2.1 Primary objective

To evaluate the effectiveness of Tuina versus standardized usual care in reducing fatigue severity in patients with chronic fatigue syndrome, based on the change in FS-14 total score from baseline to week 8.

### 2.2 Secondary clinical objectives

To assess the effects of Tuina on quality of life, sleep, pain, emotional symptoms, gastrointestinal symptoms, bowel function, and handgrip strength.

### 2.3 Exploratory mechanistic objective

To explore whether clinical improvement is accompanied by changes in tryptophan-pathway metabolites, gut microbiota composition, and brain functional and neurochemical measures, thereby generating hypotheses regarding potential gut-brain mechanisms of Tuina in CFS.

### 2.4 Hypotheses

We hypothesize that, compared with standardized usual care, Tuina will produce greater improvement in fatigue severity and related clinical outcomes in patients with CFS. We further hypothesize that these clinical changes may be accompanied by alterations in tryptophan-pathway metabolites, gut microbiota composition, and brain functional or neurochemical measures, consistent with a modulating effect on gut-brain mechanisms relevant to CFS.

## 3 Methods and analysis

### 3.1 Study design

**Figure 1:**
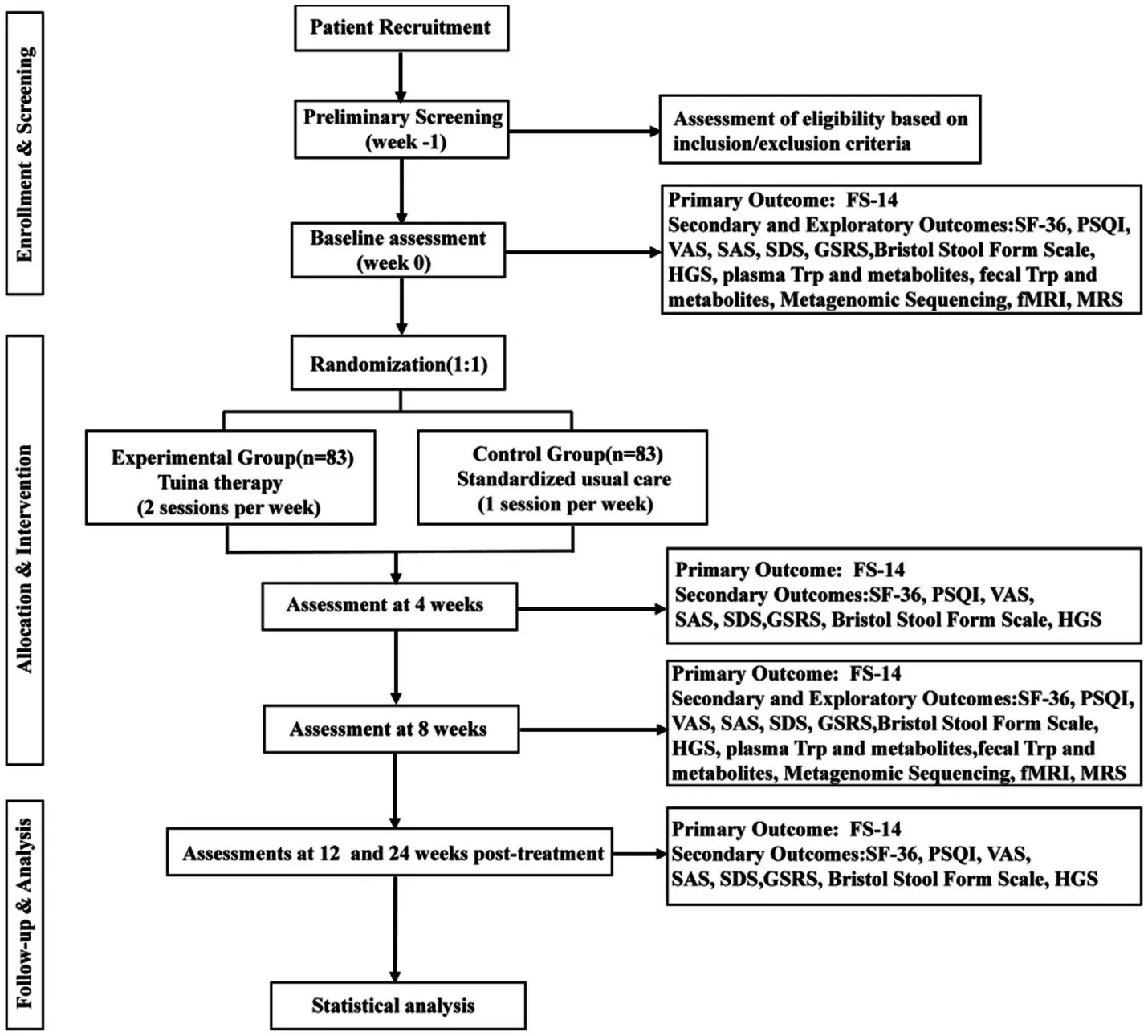
The flow chart of this trial.

This study is a multicenter, parallel-group, open-label randomized controlled trial with blinded outcome assessors and statisticians. The trial is organized with Shuguang Hospital Affiliated to Shanghai University of Traditional Chinese Medicine serving as the primary study center and Yueyang Hospital of Integrated Traditional Chinese and Western Medicine participating as an additional recruiting center. The study protocol has been approved by the Ethics Committee of Shuguang Hospital Affiliated to Shanghai University of Traditional Chinese Medicine and by the Ethics Committee of Yueyang Hospital of Integrated Traditional Chinese and Western Medicine, and has been registered at the International Traditional Medicine Clinical Trial Registry (ITMCTR2025002204). Participants will be randomly allocated in a 1:1 ratio to either the Tuina group or the control group (standardized usual care), and both groups will receive their assigned interventions for 8 weeks. Outcome assessments will be conducted at baseline, week 4, week 8 (end of treatment), and 12 and 24 weeks after the end of treatment. To improve participant retention and acknowledge trial participation, participants in the control group will be offered two Tuina sessions after completion of the final 24-week follow-up assessment. This protocol was developed in accordance with the Standard Protocol Items: Recommendations for Interventional Trials (SPIRIT) statement (Chan et al., 2025). The study flowchart is presented in Figure 1, and the detailed schedule of enrollment, interventions, and assessments is shown in Table 1.

**Table 1:** Study schedule.

Time points	Study period					
	Enrollment	Treatment			Follow-up	
	Week −1	Week 0	Week 4	Week 8	12 weeks post-treatment	24 weeks post-treatment
Enrollment						
Eligibility screen	✓					
Informed consent	✓					
Demographic characteristics	✓					
Medical history	✓					
Allocation		✓				
Physical Examination	✓					
Comorbidities	✓					
Interventions						
Tuina		✓	✓	✓		
Standardized usual care		✓	✓	✓		
Outcomes						
FS-14		✓	✓	✓	✓	✓
SF-36		✓	✓	✓	✓	✓
Pain-VAS		✓	✓	✓	✓	✓
PSQI		✓	✓	✓	✓	✓
SAS		✓	✓	✓	✓	✓
SDS		✓	✓	✓	✓	✓
GSRS		✓	✓	✓	✓	✓
BSFS		✓	✓	✓	✓	✓
HGS		✓	✓	✓	✓	✓
fMRI		✓		✓		
MRS		✓		✓		
Blood and fecal metabolites		✓		✓		
Metagenomic Sequencing		✓		✓		
Assessments						
Patient compliance		✓	✓	✓		
Adverse events		✓	✓	✓		
Concomitant medications		✓	✓	✓	✓	✓

FS-14, Fatigue Scale-14; SF-36, 36-Item Short Form Health Survey; Pain-VAS, Visual Analog Scale of Pain; PSQI, Pittsburgh Sleep Quality Index; SAS, Self-Rating Anxiety Scale; SDS, Self-Rating Depression Scale; GSRS, Gastrointestinal Symptom Rating Scale; BSFS, Bristol Stool Form Scale; HGS, Handgrip strength; fMRI, functional Magnetic Resonance Imaging; MRS, Magnetic Resonance Spectroscopy; Weeks 0, 4, and 8 are counted from baseline; week 8 corresponds to the end-of-treatment assessment. Follow-up assessments are scheduled at 12 and 24 weeks after completion of treatment.

### 3.2 Participant recruitment

Participants for the randomized trial will be recruited through Shuguang Hospital Affiliated to Shanghai University of Traditional Chinese Medicine and Yueyang Hospital of Integrated Traditional Chinese and Western Medicine. Recruitment strategies will include posting study notices on the hospital website and official WeChat account, displaying posters and flyers in outpatient waiting areas and community locations, and disseminating study information through WeChat outreach. Interested individuals may complete an initial prescreening questionnaire by telephone or via a secure WeChat form, including self-reported symptoms, medical history, and basic demographic information. This remote prescreening process is intended to facilitate participation for individuals with transportation constraints.

Individuals who appear to meet the preliminary eligibility criteria will be invited to an on-site screening visit at the participating center at which they are recruited. At this visit, a designated study physician will provide the participant information sheet, obtain written informed consent, and assess eligibility. Participants who meet all eligibility criteria will then complete the baseline assessment and proceed to randomization. A screening log will be maintained to document all contacts, eligibility decisions, and reasons for exclusion. During screening, alternative clinical explanations for fatigue, including post-COVID condition when clinically relevant, will be considered by the study physician in determining trial eligibility.

### 3.3 Eligibility criteria

Inclusion criteria:

1) The diagnostic criteria of potential participants refer to the 2015 Institute of Medicine (IOM) criteria for CFS (Clayton, 2015):

- Substantial reduction or impairment in activity level with fatigue >6 months,
- Post-exertional malaise (PEM),
- Unrefreshing sleep,
- One of the symptoms: cognitive impairment or orthostatic intolerance.

2) Age 20–60 years, of either sex, and right-handed.
3) No use within the past month of treatments considered likely to affect the primary outcomes or mechanistic assessments, including herbal formulas, acupuncture, other related non-pharmacological therapies, and antibiotics, probiotics, or prebiotics.
4) Able to communicate and complete study questionnaires, and willing to accept randomization and provide written informed consent.

Exclusion criteria:

1. Fatigue symptoms that do not lead to a substantial reduction in the capacity for work, education, social and recreational activities, or activities of daily living.
2. Severe comorbid conditions, including decompensated cardiac, cerebrovascular, hepatic or renal disease, malignancy, active infection, or neurological disorders.
3. Severe psychiatric disorder, or severe depressive symptoms requiring immediate treatment, as indicated by a Self-Rating Depression Scale score ≥63 or clinical judgment.
4. Pregnancy, planned pregnancy or lactation.
5. Contraindications to MRI, such as pacemaker, neurostimulator, metallic implants or insulin pumps, in participants enrolled in the imaging substudy.
6. Morbid obesity, defined as BMI > 45 kg/m^2^.
7. Any other condition that, in the opinion of the investigator, would contraindicate participation or continuation in the trial, or would provide a more likely alternative clinical explanation for fatigue.

### 3.4 Sample size calculation

The sample size was determined for the primary outcome, defined as the between-group difference in change in Fatigue Scale-14 (FS-14) score from baseline to week 8. Based on pilot data, the expected mean difference between groups was 1.60 points, with a pooled SD of 3.28. Using a two-sided alpha of 0.05 and 80% power for a superiority design, the required sample size was 66 participants per group. Assuming a 20% dropout rate, the final target sample size was set at 83 participants in each group, giving a total of 166 participants. The calculation was based on the standard formula for comparing two parallel groups and confirmed using G*Power (v3.1) and R (v4.3.3).

### 3.5 Randomization and blinding

Randomization will be performed using block randomization with randomly varying block sizes of 4 and 6. The allocation sequence will be generated independently by a statistician using R (version 4.3.3). To maintain balance across the two participating sites, randomization will be stratified by study center. Sequentially numbered, sealed, opaque envelopes containing the group assignments will be prepared in advance and kept by an independent trial coordinator. After eligibility has been confirmed and baseline assessments completed, the coordinator will open the next envelope in sequence to reveal the allocation. The allocation sequence will be centrally generated and maintained by an independent coordinator not involved in recruitment. Because Tuina is delivered as a manual intervention, neither therapists nor participants can be blinded to treatment assignment. However, outcome assessors, laboratory personnel, imaging analysts, data managers, and statisticians will remain masked to group allocation wherever applicable. Unblinding will occur only after database lock or when required for safety reasons. Although baseline fatigue severity is clinically important, it was not included as a stratification factor in the final randomization scheme in order to maintain consistency of the allocation procedure throughout participant enrollment.

Questionnaire-based outcomes will be collected by assessors who are not involved in treatment delivery and who will remain unaware of group allocation. Biological samples will be labeled with coded study identifiers before laboratory processing, and laboratory personnel performing metabolomic and microbiome analyses will remain blinded to treatment assignment. Imaging data will be de-identified and coded before preprocessing and analysis, and imaging analysts will work with masked group labels until completion of the predefined preprocessing and analytic steps. Final statistical analyses will be conducted using masked group labels, with unblinding only after database lock unless required for participant safety or other prespecified operational reasons.

### 3.6 Interventions

#### 3.6.1 Experimental group

Participants in the experimental group will receive the Tuina intervention in addition to the general trial advice provided to all participants, including maintaining their usual activity level and refraining from initiating any new CFS-related therapies during the 8-week treatment period. The Tuina intervention will be delivered twice weekly for 8 weeks (16 sessions in total), with a minimum interval of 48 h between sessions. Each session will last approximately 20 min. Any concomitant treatments and changes in medical care during the study period will be recorded in the case report forms (CRFs).

The Tuina therapy will focus on the abdomen (Conception vessel and key abdominal acupoints), back (the first line of the Bladder meridian and Back-Shu points), and lumbar region, with the aim of influencing autonomic regulation and gastrointestinal function. The intervention will be delivered by six licensed Tuina therapists, each with at least 5 years of clinical experience. Before participant enrollment, all therapists will receive unified training in the study-specific standard operating procedures.

A typical 20-min treatment session will follow a standardized sequence. In the supine position, one-finger pushing will be applied to Zhongwan (CV12), Qihai (CV6), and Tianshu (ST25) for 1 min at each point, followed by palm rubbing over the abdomen for 5 min until local warmth is achieved. Kneading will then be performed at CV12, CV6, and ST25 for 1 min at each point, followed by pressing-kneading at Zusanli (ST36) and Sanyinjiao (SP6) for 1 min at each point. In the prone position, one-finger pushing and rolling will be applied along the first line of the Bladder meridian on the back, with emphasis on the Back-Shu points of the five zang organs, including Feishu (BL13), Xinshu (BL15), Ganshu (BL18), Pishu (BL20), and Shenshu (BL23). Linear rubbing will then be performed along the Governor vessel and Bladder meridian until local warmth is achieved. Acupoints will be located in accordance with GB/T 12346–2021 (Naming and Positioning of Acupoints). Representative images of the standardized Tuina procedures are provided in Supplementary Figure 1. Detailed acupoint information and intervention procedures are provided in Supplementary Table 1.

#### 3.6.2 Control group

The control group will receive standardized usual care. Participants in the control group will receive health education and symptom-oriented management once weekly for 8 weeks, with each session lasting approximately 40 min. The program will cover balanced diet, sleep hygiene, stress management, psychological support, and tailored symptom management advice when clinically indicated. To reduce contamination, additional interventions specifically targeting fatigue, such as newly initiated pharmacotherapy, acupuncture, or herbal prescriptions, will not be permitted during the 8-week treatment period unless deemed clinically necessary. Stable pre-existing medications may be continued. Standardized usual care will be delivered by two TCM attending physicians who are licensed clinicians and have completed study-specific training and Good Clinical Practice certification. Concomitant medications and any rescue treatments will be recorded at each visit. Staff qualifications, training records, and adherence to the intervention protocol will be documented and audited.

Participant blinding is not feasible because of the nature of the Tuina intervention. The control group will receive health education and symptom-oriented management with standardized contact procedures and approximately matched total contact time, which is intended to help reduce imbalances in attention and trial engagement between groups. However, because treatment frequency and tactile contact cannot be matched, residual differences in treatment expectancy and performance effects may remain. This comparator was chosen to reflect current clinical management for CFS, in which symptom-oriented and supportive care remains the predominant non-pharmacological management approach. The study therefore evaluates standardized Tuina in comparison with standardized usual care, rather than against an inert, no-treatment, or fully attention-matched sham condition.

### 3.7 Outcomes

#### 3.7.1 Primary outcome

The primary outcome is fatigue severity, assessed as the change in Fatigue Scale-14 (FS-14) total score from baseline to week 8. The FS-14 is a patient-reported instrument comprising two domains: physical fatigue (items 1–8) and mental fatigue (items 9–14) (Chalder et al., 1993). Items 10, 13, and 14 are reverse-worded and will be recorded according to the scoring manual so that higher scores indicate greater fatigue. For the primary analysis, binary scoring (0–1 per item) will be used, yielding a total score ranging from 0 to 14, with subscale scores ranging from 0 to 8 for physical fatigue and 0 to 6 for mental fatigue. The FS-14 has demonstrated acceptable validity and reliability in Chinese populations (Wang et al., 2000). It will be administered at baseline, week 4, week 8, and at follow-up visits at weeks 12 and 24 post-treatment. Participants will complete the questionnaire with reference to their experience during the preceding week.

FS-14 was selected as the primary outcome because fatigue severity remains the most clinically prominent and pragmatically measurable symptom domain in CFS trials. Post-exertional malaise, a core clinical feature of CFS, will be further assessed at baseline using the DePaul Symptom Questionnaire–Post-Exertional Malaise (DSQ-PEM) as part of participant characterization.

#### 3.7.2 Secondary outcomes

Health-related quality of life will be assessed using the 36-Item Short Form Health Survey (SF-36) (Ware and Sherbourne, 1992). The SF-36 covers eight domains: physical functioning, role physical, bodily pain, general health, vitality, social functioning, role emotional, and mental health. Each domain score will be transformed to a 0–100 scale, with higher scores indicating better health status.

Sleep quality will be assessed using the Pittsburgh Sleep Quality Index (PSQI) (Buysse et al., 1989). The PSQI includes 19 self-rated items that generate seven component scores: subjective sleep quality, sleep latency, sleep duration, habitual sleep efficiency, sleep disturbances, use of sleep medication, and daytime dysfunction. The global score ranges from 0 to 21, with higher scores indicating poorer sleep quality.

Pain intensity will be assessed using a 10-cm visual analog scale (VAS) (Hawker et al., 2011), ranging from 0 (no pain) to 10 (worst imaginable pain), with higher scores indicating greater pain intensity.

Anxiety and depressive symptoms will be assessed using the Self-Rating Anxiety Scale (SAS) and Self-Rating Depression Scale (SDS), respectively (Zung, 1971; Zung, 1965). Each scale contains 20 items scored from 1 to 4, yielding a raw total score of 20–80. In accordance with standard scoring procedures, raw scores will be multiplied by 1.25 to obtain index scores ranging from 25 to 100. Higher scores indicate greater symptom severity.

Gastrointestinal symptoms will be assessed using the Gastrointestinal Symptom Rating Scale (GSRS) (Revicki et al., 1998). The GSRS comprises 15 items scored on a 7-point Likert scale and evaluates the severity of common gastrointestinal symptoms, including reflux, abdominal pain, indigestion, diarrhea, and constipation-related symptoms. Higher scores indicate more severe gastrointestinal symptoms.

Bowel function will be assessed using the Bristol Stool Form Scale (BSFS) and a stool frequency diary (Lewis and Heaton, 1997). The BSFS classifies stool form into seven categories, with types 1–2 indicating constipation and types 3–5 indicating normal stool form.

Handgrip strength (HGS) testing will be used to assess muscle strength and fatigability (Popkirov, 2025). Participants will perform 10 consecutive maximal grips using a hand dynamometer and will repeat the same procedure 60 min later. For each session, maximal force (Fmax) and mean force (Fmean), expressed in kilograms, will be recorded. The first session values will be denoted as Fmax1 and Fmean1, and the second session values as Fmax2 and Fmean2. The fatigue ratio will be calculated as Fmax/Fmean, with higher values indicating a greater decline in force across repeated contractions (Jäkel et al., 2021).

#### 3.7.3 Exploratory mechanistic outcomes

Exploratory mechanistic assessments will be conducted according to predefined sampling frameworks. Plasma metabolomic analyses and stool metagenomic analyses will be attempted in all randomized participants who provide usable biological samples at the specified time points (baseline and week 8). Resting-state fMRI and MRS will be conducted in a predefined imaging substudy composed of participants who provide additional consent and have no MRI contraindications. Participation in the imaging substudy is optional and is not required for enrollment in the main randomized trial. We expect that approximately 60 participants of the full cohort will participate in the imaging substudy, depending on consent, eligibility, and scanner availability. These exploratory mechanistic assessments are not formally powered for confirmatory hypothesis testing and are intended to estimate effect sizes, characterize biological patterns, and generate hypotheses for future studies.

The rationale for each exploratory domain is described below. First, disturbances in tryptophan metabolism, particularly within the kynurenine pathway, have been implicated in immune activation, neuroactive metabolite imbalance, and fatigue-related symptoms. Alterations in the KYN/Trp ratio may reflect inflammatory signaling and indoleamine 2,3-dioxygenase activity (Muneer, 2020). Recent CFS studies have suggested abnormal tryptophan catabolism and increased kynurenine production, indicating that tryptophan metabolites may link peripheral dysregulation with central symptoms such as cognitive dysfunction and sleep disturbance (Groven et al., 2021; Hsu et al., 2025). Accordingly, plasma and stool samples will be collected to quantify tryptophan and its downstream metabolites using targeted liquid chromatography-mass spectrometry (LC–MS) (Desmons et al., 2022). Samples will be processed according to standardized laboratory procedures, as described in the online Supplementary materials.

Second, gut microbiota will be evaluated as an exploratory outcome. Growing evidence supports an association between CFS and gut microbial dysbiosis, with reported alterations in microbial composition, barrier-related processes, and microbially mediated metabolites (Wang et al., 2024; Prylińska-Jaśkowiak et al., 2025; El-Sehrawy et al., 2025; Stallmach et al., 2024). However, current evidence remains insufficient to establish a causal role of the gut microbiota in CFS pathogenesis, underscoring the need for integrative clinical studies linking microbial measures with symptoms and brain-based outcomes. Because Tuina is applied over the abdomen and paraspinal regions and may influence autonomic regulation and gastrointestinal function, stool metagenomic profiling will be used to explore whether the intervention is associated with changes in gut microbial composition and function. Stool samples will be collected at predefined time points, and metagenomic sequencing will be conducted using standardized procedures. Detailed methods for library preparation, sequencing, and bioinformatic analysis are provided in the online Supplementary materials.

Third, fMRI and MRS will be used to explore potential central mechanisms of Tuina in CFS. Resting-state fMRI studies in CFS have reported altered functional connectivity within and between large-scale brain networks related to fatigue, pain, and cognitive symptoms (Su et al., 2023; Boissoneault et al., 2016). Proton MRS studies have also identified abnormalities in brain metabolites associated with energy metabolism and neuroinflammatory processes (Godlewska et al., 2025; Mueller et al., 2020). Magnetic resonance spectroscopy offers an *in vivo* window into neurochemical processes potentially relevant to fatigue-related disorders. Recent ultra-high-field MRS work in CFS has identified elevated anterior cingulate lactate, consistent with energetic stress and altered mitochondrial-related metabolism, supporting the inclusion of MRS as a complementary modality alongside rs-fMRI in mechanistic studies of CFS (Godlewska et al., 2025). In this study, resting-state fMRI and MRS will be acquired on a 3.0 T Siemens scanner using standardized protocols. Functional imaging analyses will focus on amplitude of low-frequency fluctuation, regional homogeneity, and functional connectivity, while MRS analyses will examine region-specific metabolite profiles in predefined brain regions of interest. Detailed acquisition parameters, preprocessing procedures, motion-control criteria, and region-of-interest definitions are provided in the online Supplementary materials.

### 3.8 Safety monitoring

AEs will be assessed at each treatment session and at each study contact during the intervention period. Their severity, duration, relationship to the intervention, actions taken, and outcomes will be documented in the case report form (CRF). Potential intervention-related adverse events may include transient soreness, skin irritation, or bruising at treatment sites. If a participant experiences an adverse reaction, appropriate medical care will be provided promptly, and the event will be recorded in detail in the CRF. Decisions regarding treatment interruption or withdrawal from the study will be made by the principal investigator in accordance with the study protocol and institutional requirements. Serious adverse events (SAEs) will be documented immediately, assessed for severity and relatedness by the principal investigator, and reported to the ethics committee in accordance with institutional requirements.

### 3.9 Statistical analysis

Statistical analyses will be performed using SPSS version 26.0 (IBM Corp., Armonk, NY, USA). Baseline characteristics will be summarized as mean (SD) or median (IQR) for continuous variables and as number (%) for categorical variables, as appropriate. Longitudinal outcomes will be analyzed using linear mixed-effects models including fixed effects for group, time, and group-by-time interaction. The models will be adjusted for baseline outcome values, study center, and sex, with additional prespecified covariates included where appropriate. For the primary outcome analysis, baseline FS-14 score will be included as a covariate. Secondary clinical outcomes will be interpreted with consideration of multiplicity, and exploratory omics and imaging analyses will be interpreted as hypothesis-generating. All tests will be two-sided, and a *p* value <0.05 will be considered statistically significant. The primary efficacy analysis will follow the intention-to-treat principle. A per-protocol analysis will be conducted as a sensitivity analysis. The primary comparison will be the between-group difference in change in FS-14 total score from baseline to week 8, estimated from the mixed-effects model. Missing data will be handled under the missing-at-random assumption inherent to mixed-effects models, and additional sensitivity analyses will be conducted if warranted. Because this is a multicenter trial, study center will be included in the analysis model to account for potential between-center variability. All statistical analyses will be performed with group allocation concealed until database lock. The full trial protocol is provided in this article and its online Supplementary materials.

### 3.10 Metabolomics and microbiota analyses

These analyses are exploratory and hypothesis-generating, are not formally powered for confirmatory inference, and will be interpreted primarily as effect-size estimation and biological signal characterization rather than definitive hypothesis testing. These findings will not be interpreted as confirmatory efficacy evidence. Targeted metabolomics data will be analyzed using multivariate and univariate approaches to identify differential metabolites, with particular focus on the tryptophan pathway. Multivariate analyses will include principal component analysis and supervised discrimination methods, whereas univariate comparisons will be corrected for multiple testing using the false discovery rate (FDR). Pathway enrichment analyses will then be performed, and associations between metabolite changes and clinical outcomes will be examined using Pearson or Spearman correlation analyses, as appropriate.

Microbiota data will be analyzed at the levels of alpha diversity, beta diversity, and differential taxonomic abundance. Alpha diversity indices will be compared using appropriate parametric or nonparametric methods according to data distribution and study design. Beta diversity will be assessed using permutational multivariate analysis of variance, and differentially abundant taxa will be identified using FDR-controlled methods. Associations between microbial features and clinical measures will be explored using multivariable regression and ordination-based approaches, as appropriate.

### 3.11 Imaging preprocessing and analysis

Imaging data will be processed using a standardized pipeline in MATLAB with SPM12- and DPABI-based tools. The first 10 volumes of each run will be discarded to allow for signal stabilization. Preprocessing will include slice-timing correction, realignment for head-motion correction, coregistration to each participant’s structural T1-weighted image, normalization to Montreal Neurological Institute space, resampling, and spatial smoothing. Structural images will be segmented into gray matter, white matter, and cerebrospinal fluid and normalized using established procedures. Nuisance regression will include motion parameters and signals from white matter and cerebrospinal fluid, followed by detrending and band-pass filtering. Volumes exceeding predefined motion thresholds will be censored, and participants exceeding prespecified motion criteria will be excluded. The specific thresholds for framewise displacement, translational/rotational motion, and participant exclusion are provided in the online Supplementary materials. Detailed MRI acquisition parameters, preprocessing procedures, motion criteria, and quality-control steps are provided in the online Supplementary materials.

### 3.12 Data management and monitoring

CRFs will be completed by trained study staff. Two independent data managers, blinded to treatment allocation, will perform double data entry and verify the study database. Source documents will be stored securely at the study site, and electronic data will be maintained on password-protected servers with role-based access control. Participant confidentiality will be protected by assigning unique study identification numbers and storing personal identifiers separately from research data. Only de-identified, aggregate data will be reported in publications. Shuguang Hospital Affiliated to Shanghai University of Traditional Chinese Medicine will serve as the coordinating site and will be responsible for overall trial coordination and protocol implementation. Safety oversight will be provided by the registered Data and Safety Monitoring Committee in accordance with institutional requirements. Quality assurance procedures will include site training, standardized operating procedures, regular instrument calibration, and periodic monitoring. Intervention fidelity will be supported through standardized therapist training, treatment logs, and periodic checks of protocol adherence. Treatment records will document session completion, adherence to standardized procedures, and any protocol-related variations identified during the trial. Participant adherence will be monitored by recording session attendance and reasons for missed visits and will be supported through reminder contacts and flexible scheduling within protocol limits. Lifestyle-related information that may influence microbiome and metabolic profiles, including major changes in dietary habits, physical activity patterns, and other relevant health behaviors, will be recorded when applicable. No formal interim efficacy analyses are planned. The Data and Safety Monitoring Committee may recommend trial modification or termination in the event of unacceptable safety concerns, major protocol violations, or other circumstances that materially affect participant safety or trial feasibility.

## 4 Discussion

A central challenge in the management of CFS is the lack of effective and scalable non-pharmacological therapies. To address this gap, we designed a superiority trial with blinded outcome assessment and prespecified analyses, comparing standardized Tuina with standardized usual care. Accordingly, this design is intended to compare the effects of standardized Tuina with those of standardized usual care in a clinically relevant setting, while also allowing assessment of the durability of any treatment effects over a 24-week follow-up period. At the same time, because the standardized usual care condition includes lifestyle counselling and symptom-oriented support, it may itself influence fatigue-related symptoms and, potentially, gut-related measures. Therefore, any between-group differences should be interpreted as the comparative effect of standardized Tuina versus standardized usual care, rather than as the isolated effect of Tuina against an inert control. In addition, the open-label nature of the trial should be considered when interpreting patient-reported outcomes. Because participant and therapist blinding is not feasible in this type of manual therapy study, the primary outcome (FS-14) and several secondary outcomes (including PSQI, SAS, and SDS) rely on self-reported assessments and may therefore be influenced by expectation effects, reporting bias, and participants’ awareness of treatment allocation. While blinded outcome assessment, standardized data handling, and prespecified statistical analysis plans have been implemented to reduce detection and analytical bias, these procedures cannot fully eliminate bias inherent to subjective outcome reporting in an open-label design. Accordingly, interpretation of these outcomes should be made with appropriate caution.

Beyond evaluating clinical effectiveness, the present study is intended to explore a biologically informed mechanistic framework for CFS. To our knowledge, this integrative design distinguishes the present protocol from most previous CFS trials, which have generally focused on clinical outcomes alone. This trial is also designed as an exploratory mechanistic study focusing primarily on the tryptophan-kynurenine pathway as a candidate link between gastrointestinal disturbance and central symptom expression in CFS. Increasing evidence implicates neuroendocrine–immune dysregulation and the gut-brain axis in CFS, but the relevant causal pathways remain poorly defined. Previous studies have reported altered tryptophan metabolites in cerebrospinal fluid and peripheral samples from patients with CFS, while clinical and microbiome studies have consistently noted gastrointestinal complaints and gut dysbiosis (Walitt et al., 2024; Leong et al., 2022; Gao et al., 2020). Because tryptophan and its downstream catabolites lie at the interface between gut microbial activity, immune signaling, and central nervous system function, the tryptophan–kynurenine pathway represents a biologically plausible focal mechanism linking peripheral disturbance with fatigue-related symptoms. We therefore selected plasma and stool tryptophan-pathway metabolites as the primary exploratory mechanistic domain.

Fecal metagenomic profiles and central network measures assessed by resting-state fMRI and MRS will be treated as secondary exploratory domains intended to contextualize any observed biochemical changes. This hierarchical design is intended to reduce interpretive overreach while still allowing assessment of broader gut-brain features that may be relevant to symptom improvement. This approach also reflects the differing levels of prior biological plausibility and measurement maturity across the exploratory domains. In addition, evidence from related conditions and mechanistic studies suggests that Tuina, as an external manual therapy, may influence parasympathetic activity (Goldenberg and Kalichman, 2024; Henley et al., 2008), gastrointestinal function (Geng et al., 2020; Stringer et al., 2008), microbial ecology (Sachula et al., 2024), and may also modulate brain processes relevant to fatigue, pain, and mood (Wu et al., 2023; Ma et al., 2023). However, these pathways should be regarded as biologically plausible candidate mechanisms rather than established causal effects in CFS. In this respect, the integration of omics-based and neuroimaging-based measures may help generate candidate biomarkers and responder profiles for future studies.

In summary, this study is designed not only to provide higher-quality clinical evidence for Tuina in CFS, but also to serve as a mechanistic pilot examining associations between symptom change and alterations in tryptophan metabolism, gut microbial features, and brain functional characteristics. If the trial demonstrates clinically meaningful improvement together with convergent exploratory biomarker changes, the findings will provide preliminary support for larger multicenter confirmatory studies and help refine mechanistic hypotheses regarding Tuina in CFS, particularly with respect to gut-related symptoms and gut-brain mechanisms. At the same time, the exploratory nature of the omics and imaging analyses means that these findings should be interpreted as hypothesis-generating rather than definitive evidence of causality.
