# Supplementary material for: Tuina for chronic fatigue syndrome: study protocol for a randomized controlled trial integrating tryptophan metabolism, gut microbiota, and brain network alterations
Source: Front Neurosci. 2026 Aug 12;20:1859226. doi: 10.3389/fnins.2026.1859226 (PMC13506880; doi:10.3389/fnins.2026.1859226)
Supplement: Supplementary file 1 [file Supplementary_file_1.docx]

Supplementary Figure 1. Representative procedures of the standardized Tuina intervention

|   (A) |   (B) |
| --- | --- |
|   (C) |   (D) |
| (A–D) Representative procedures performed in the supine position, including point-focused manipulation, palm rubbing over the abdomen, kneading over the abdominal region, and pressing-kneading at lower-limb acupoints | |
|   (E) |   (F) |
|   (G) |   (H) |
| (E-H) Representative procedures performed in the prone position, including point-focused manipulation, rolling over the paraspinal region, and linear rubbing over the back and lumbar region | |

Supplementary Table 1. Locations of acupoints for the standardized Tuina protocol

| Acupoint | Location |
| --- | --- |
| CV12 | On the anterior midline of the upper abdomen, 4 B-cun superior to the center of the umbilicus |
| CV6 | On the anterior midline of the lower abdomen, 1.5 B-cun inferior to the center of the umbilicus |
| ST25 | On the abdomen, 2 B-cun lateral to the center of the umbilicus, at the level of CV8 (Shenque) |
| ST36 | On the anterior aspect of the leg, on the line connecting Dubi (ST35) and Jiexi (ST41), 3 B-cun inferior to ST35, one finger-breadth lateral to the anterior crest of the tibia |
| SP6 | On the medial aspect of the leg, 3 B-cun superior to the prominence of the medial malleolus, posterior to the medial border of the tibia |
| BL13 | On the back, 1.5 B-cun lateral to the lower border of the spinous process of the 3rd thoracic vertebra (T3) |
| BL15 | On the back, 1.5 B-cun lateral to the lower border of the spinous process of the 5th thoracic vertebra (T5) |
| BL18 | On the back, 1.5 B-cun lateral to the lower border of the spinous process of the 9th thoracic vertebra (T9) |
| BL20 | On the back, 1.5 B-cun lateral to the lower border of the spinous process of the 11th thoracic vertebra (T11) |
| BL23 | On the back, 1.5 B-cun lateral to the lower border of the spinous process of the 2nd lumbar vertebra (L2) |
| Paraspinal Bladder Meridian 1st Line | On the back, running parallel to the posterior midline, 1.5 B-cun lateral to the spinous processes of the thoracic, lumbar, and sacral vertebrae, corresponding to the first line of the Bladder meridian and including the Back-Shu points |
| Epigastrium | The upper abdominal region, bounded superiorly by the xiphoid process, inferiorly by the umbilicus, and laterally by the costal margins |

Online Supplemental Materials

Supplementary Methods

1. Targeted metabolomics of tryptophan-pathway metabolites

Tryptophan-pathway metabolites will be quantified in plasma and stool samples using targeted liquid chromatography-mass spectrometry (LC-MS). Peripheral venous blood and stool samples will be collected from each participant according to standardized procedures. For plasma analysis, 50 µL of plasma will be transferred to an Eppendorf tube and mixed with internal standards. Proteins will be precipitated with three volumes of methanol–acetonitrile (1:1, v/v). After centrifugation at 15,000 rpm for 15 minutes, the supernatant will be collected, dried under vacuum, and reconstituted in 60 µL of mobile phase containing 50% organic solvent in 0.2% formic acid in water. After a second centrifugation at 15,000 rpm for 15 minutes, the final supernatant will be used for LC-MS analysis. For stool analysis, approximately 15 ± 1 mg of stool will be extracted with 100 µL of 50% methanol in water. After sonication for 15 minutes and centrifugation at 4°C, an aliquot of the supernatant will be transferred to a clean tube, mixed with internal standards, and subjected to protein precipitation with methanol–acetonitrile (1:1, v/v). Following centrifugation, the supernatant will be dried under vacuum, reconstituted in 60 µL of 50% methanol in water, and centrifuged again. The resulting supernatant will be injected for LC-MS analysis (González-Domínguez et al., 2023).

2. Metagenomic sequencing and bioinformatic processing

Metagenomic libraries will be sequenced on an Illumina NovaSeq 6000 platform to generate 150 bp paired-end reads. Raw reads in FASTQ format will be trimmed and quality-filtered using Trimmomatic (v0.36) (Bolger et al., 2014). Host-derived reads will be removed by alignment to the host genome with Bowtie2 (v2.2.9), and mapped reads will be discarded. The remaining reads will be assembled using MEGAHIT (v1.1.2) (Li et al., 2015; Li et al., 2016). Scaffolds will be split at internal gaps to generate contigs (scaftigs), and sequences longer than 500 bp will be retained. Open reading frames will be predicted using Prodigal (v2.6.3) (Hyatt et al., 2010) and translated into amino acid sequences. A nonredundant gene catalog will be generated with CD-HIT (v4.5.7) using 95% sequence identity and 90% coverage as clustering thresholds. The longest gene in each cluster will be retained as the representative sequence. Clean reads from each sample will then be aligned to the nonredundant gene set with Bowtie2, and gene abundance will be estimated from the number of mapped reads.

3. MRI acquisition

For participants enrolled in the predefined imaging substudy, MRI data will be acquired on a 3.0 T Siemens scanner with a 32-channel head coil. The imaging protocol will include structural MRI, resting-state functional MRI (rs-fMRI), and proton magnetic resonance spectroscopy (MRS), where applicable. For rs-fMRI, blood oxygen level-dependent images will be acquired using gradient-echo echo-planar imaging with the following parameters: repetition time 2000 ms, echo time 30 ms, flip angle 90°, matrix size 64×64, 33–40 axial slices, voxel size 3.5×3.5×3.5 mm³, and 240 volumes. Total acquisition time will be approximately 8 minutes. Participants will be asked to arrive 30 minutes prior to scanning and to rest quietly for 10 minutes before scanning begins. During rs-fMRI acquisition, they will be instructed to keep their eyes closed, stay awake, avoid active thinking, and minimize head movement. Foam padding and a head holder will be used to restrict motion, and earplugs will be provided to reduce scanner noise. All scans will be performed by an experienced MRI technologist who will not be involved in outcome assessment. Structural T1-weighted images will be acquired for anatomical reference and spatial normalization. Proton MRS will be performed using a PRESS-CSI sequence with a repetition time of 4280 ms and an echo time of 135 ms. Water suppression will be achieved using chemical shift selective suppression after automated higher-order B0 shimming, and outer-volume suppression bands will be applied to reduce lipid contamination (Zhu and Barker, 2011). The spectroscopic field of view will be 230×230×230 mm³ with a slab thickness of 5 mm and an interslice gap of 1.5 mm. A non-suppressed water reference scan will be acquired for eddy-current correction and signal scaling. One slab will be positioned over the cerebellum, with voxels indexed 1–16 in the right cerebellar hemisphere, 17–24 in the vermis, and 25–40 in the left cerebellar hemisphere. A second slab will be centered on the anterior cingulate cortex and aligned to the anterior commissure-posterior commissure plane.

4. MRI preprocessing and derivation of imaging measures

Imaging data will be preprocessed in MATLAB using SPM12, DPABI, and CAT12. For rs-fMRI, the first 10 volumes of each run will be discarded to allow for signal stabilization. Preprocessing will include slice-timing correction, realignment for head-motion correction, coregistration to the corresponding structural T1-weighted image, spatial normalization to Montreal Neurological Institute space, resampling, and spatial smoothing. Structural images will be segmented into gray matter, white matter, and cerebrospinal fluid using SPM12/CAT12 and normalized with DARTEL-based procedures. For voxel-based morphometry, modulated gray-matter maps will be smoothed with an 8 mm full-width at half-maximum Gaussian kernel. For functional preprocessing, normalized rs-fMRI images will be resampled to 3×3×3 mm³ isotropic voxels and smoothed with a 6 mm full-width at half-maximum Gaussian kernel. Nuisance regression will include the Friston 24-parameter head-motion model and mean white matter and cerebrospinal fluid signals. Linear detrending and temporal band-pass filtering (0.01–0.08 Hz) will then be applied. Participants will be excluded if maximum head motion exceeds 2.0 mm translation in any direction, 2.0° rotation in any plane, or a mean framewise displacement of 0.3 mm. Volumes with framewise displacement greater than 0.5 mm will be censored where applicable. After preprocessing, amplitude of low-frequency fluctuation, regional homogeneity, and functional connectivity maps will be derived using standard DPABI procedures. Regional homogeneity will be calculated using Kendall’s coefficient of concordance across 26 neighboring voxels. Seed-based functional connectivity analyses will be performed using predefined regions of interest from the Automated Anatomical Labeling atlas. Quality control will be performed at each preprocessing step through visual inspection and motion assessment before statistical analysis.

References:

González-Domínguez, Á., Armeni, M., Savolainen, O., Lechuga-Sancho, A.M., Landberg, R., González-Domínguez, R. (2023). Untargeted metabolomics based on liquid chromatography-mass spectrometry for the analysis of plasma and erythrocyte samples in childhood obesity. Methods Mol. Biol. 2571, 115–122.

Bolger, A. M., Lohse, M., and Usadel, B. (2014). Trimmomatic: a flexible trimmer for Illumina sequence data. Bioinformatics 30, 2114–2120. doi: 10.1093/bioinformatics/btu170.

Li, D., Liu, C. M., Luo, R., Sadakane, K., and Lam, T. W. (2015). MEGAHIT: an ultra-fast single-node solution for large and complex metagenomics assembly via succinct de Bruijn graph. Bioinformatics 31, 1674–1676. doi: 10.1093/bioinformatics/btv033.

Li, D., Luo, R., Liu, C. M., Leung, C. M., Ting, H. F., Sadakane, K., et al. (2016). MEGAHIT v1.0: A fast and scalable metagenome assembler driven by advanced methodologies and community practices. Methods 102, 3–11. doi: 10.1016/j.ymeth.2016.02.020.

Hyatt, D., Chen, G. L., Locascio, P. F., Land, M. L., Larimer, F. W., and Hauser, L. J. (2010). Prodigal: prokaryotic gene recognition and translation initiation site identification. BMC Bioinformatics 11, 119. doi: 10.1186/1471-2105-11-119.

Zhu, H., and Barker, P. B. (2011). MR spectroscopy and spectroscopic imaging of the brain. Methods Mol. Biol. 711, 203–226. doi: 10.1007/978-1-61737-992-5_10.
